# Understanding the Causes and Implications of Endothelial Metabolic Variation in Cardiovascular Disease through Genome-Scale Metabolic Modeling

**DOI:** 10.3389/fcvm.2016.00010

**Published:** 2016-04-18

**Authors:** Sarah McGarrity, Haraldur Halldórsson, Sirus Palsson, Pär I. Johansson, Óttar Rolfsson

**Affiliations:** ^1^Center for Systems Biology, University of Iceland, Reykjavik, Iceland; ^2^Department of Pharmacology and Toxicology, School of Health Sciences, University of Iceland, Reykjavik, Iceland; ^3^Sinopia Biosciences Inc., San Diego, CA, USA; ^4^Section for Transfusion Medicine, Capital Region Blood Bank, Rigshospitalet, University of Copenhagen, Copenhagen, Denmark; ^5^Department of Biochemistry and Molecular Biology, School of Health Sciences, University of Iceland, Reykjavik, Iceland

**Keywords:** endothelium, metabolism, personalized/precision medicine, metabolomics, metabolic modeling, genetics

## Abstract

High-throughput biochemical profiling has led to a requirement for advanced data interpretation techniques capable of integrating the analysis of gene, protein, and metabolic profiles to shed light on genotype–phenotype relationships. Herein, we consider the current state of knowledge of endothelial cell (EC) metabolism and its connections to cardiovascular disease (CVD) and explore the use of genome-scale metabolic models (GEMs) for integrating metabolic and genomic data. GEMs combine gene expression and metabolic data acting as frameworks for their analysis and, ultimately, afford mechanistic understanding of how genetic variation impacts metabolism. We demonstrate how GEMs can be used to investigate CVD-related genetic variation, drug resistance mechanisms, and novel metabolic pathways in ECs. The application of GEMs in personalized medicine is also highlighted. Particularly, we focus on the potential of GEMs to identify metabolic biomarkers of endothelial dysfunction and to discover methods of stratifying treatments for CVDs based on individual genetic markers. Recent advances in systems biology methodology, and how these methodologies can be applied to understand EC metabolism in both health and disease, are thus highlighted.

## Introduction

Cardiovascular disease (CVD) includes acute and chronic conditions, such as stroke and coronary heart disease (1). CVD results in a shortened life span and is the biggest cause of death worldwide (1–3). The endothelium is the single cell layer that lines blood vessels and lymphatic system and its dysfunction contributes to the development of CVD (4, 5). Endothelial cells (ECs) play an important role in controlling vascular tone and by secreting or expressing surface molecules, they ensure appropriate regulation of blood flow, counteracting intravascular activation of platelets, and coagulation (6, 7). Moreover, cardiac ECs have been shown to affect the ventricular myocardium. Thus, the force-frequency response of cardiac muscle in the presence of increased cardiac workload is blunted after damage to the cardiac endothelium (8).

A vascular surface that normally is thromboresistant, anti-inflammatory, vasodilatory, and antiproliferative can turn into a surface that is thrombogenic, proinflammatory, vasoconstricive, and stimulatory of smooth muscle cell proliferation. Often this change is reactive and transient restoring vascular homeostasis. However, in diseases such as atherosclerosis, hypertension, and diabetes mellitus (DM) such changes, known as endothelial dysfunction, may be prolonged and critical for disease progression. The extent of pathological metabolic perturbation is determined by an interaction of lifestyle factors, such as diet and exercise with underlying genetic factors (9–12). Consequently, health-care interventions may be more effective if adapted to an individual.

Metabolic modeling offers insights into cellular metabolism (13). Below, we consider endothelial metabolic alterations, their contribution to endothelial dysfunction, and integrated analysis of this information with genome-scale metabolic models (GEMs) to advance personalized health care.

## Endothelial Metabolism

**Figure 1:**
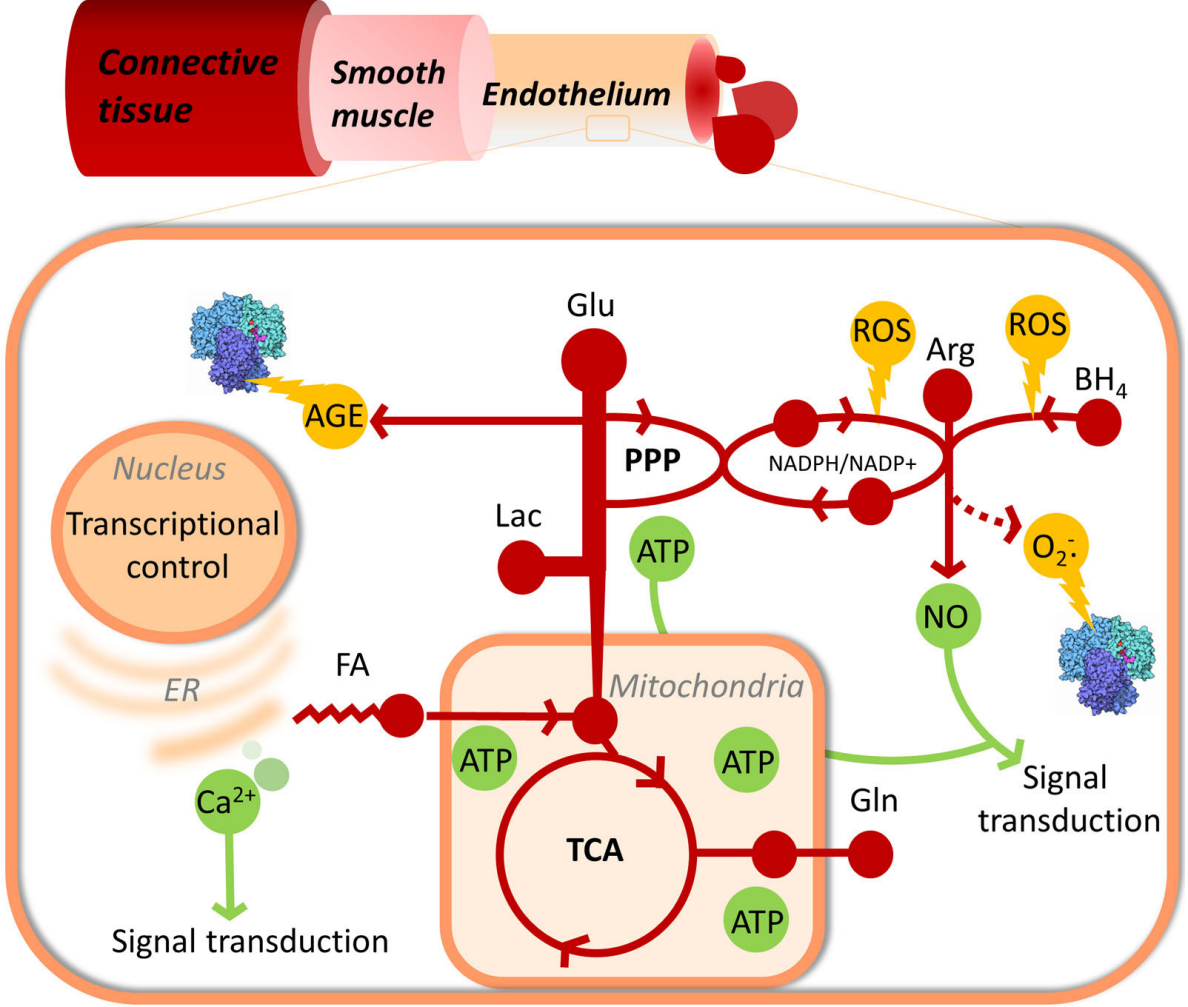
**Endothelial metabolism and its links to cellular damage, function, and proliferation control**. Metabolism, including glycolysis, pentose phosphate pathway, TCA cycle, fatty acid oxidation, and nitric oxide synthase are represented in red. Useful products of metabolism, NO, ATP, and Ca^2+^ signaling are shown in green. Damaging side products of metabolism are shown in yellow.

Endothelial cell metabolism has been investigated in multiple contexts including angiogenesis, hypoxia, shear stress, glycemia, and response to perturbations with mediators of vascular health including thrombin, sphingosine-1-phosphate, and more (14–19). The endothelium operates with variable nutrient availability and oxygen partial pressures in a manner that is EC subtype specific (20) and results in altered synergy in the oxidation of its core nutrients glucose, fatty acids, and amino acids (17, 21–23) that are reviewed specifically elsewhere (24, 25) but considered collectively here and illustrated in Figure 1.

### Glycolysis Affects Endothelial Proliferation and Angiogenesis

Endothelial cells oxidize glucose largely by glycolysis, allowing maximal availability of oxygen for transendothelial transport to perivascular cells (26–29). Carbons from glucose are primarily excreted as lactate with only 1 in 200 pyruvate equivalents contributing to oxidative phosphorylation (26). Laminar shear stress, the frictional force created by blood flow, promotes anti-inflammatory, anti-thrombotic, and anti-oxidative properties in ECs and helps to maintain quiescence largely *via* the transcription factor Kruppel-like factor 2 (30) that acts to repress phosphofructokinase-2/fructose-2,6-bisphosphatase-3 (PFKFB3) thereby promoting a quiescent phenotype (16).

In response to angiogenic factors induced by injury or in pathological conditions such as hypoxia, nutrient deprivation, or tissue damage, ECs quickly form new vasculature by sprouting. During vessel sprouting, glycolysis is increased further, mediated by increased activity of PFKFB3, the loss of which impairs vessel formation (26). Increased glycolysis without oxidation of pyruvate relies on lactate dehydrogenase to supply NAD^+^, and the activity of PFKFB3 is reflected in both intracellular and secreted lactate of ECs (31). Furthermore, lactate is involved in PFKFB3-mediated endothelial proliferation, tube formation, and Akt activation providing a plausible explanation for PFKFB3-mediated angiogenesis (31). Lactate dehydrogenase activity also increases with EC subtype proliferation rate. In pulmonary microvascular ECs, rapid angiogenesis is dependent on lactate dehydrogenase A expression (14).

Endothelial-dependent vascular function correlates with blood glucose levels (32–37). In hyperglycemia, glyceraldehyde-3-phosphate dehydrogenase is inactivated, impeding glycolysis (38). A build-up of fructose-6-phosphate, a glycolytic intermediate, impacts hexosamine biosynthesis generating *N*-acetylglucosamine that glycosylates and modifies angiogenic proteins including Notch and vascular endothelial growth factor receptor 2 (39–45) and, inhibits eNOS (46). Excess glucose also enters the polyol pathway, producing excess advanced glycation end products (AGEs) (47, 48). AGEs alter the binding of erythrocytes and platelets to the endothelium (49, 50), and clinical arterial responsiveness correlates negatively with the ratio of AGEs to soluble receptor of AGEs (51).

### Fatty Acid and Amino Acids Metabolism

Fatty acid-binding protein 4 (FABP4) is an intracellular fatty acid chaperone protein that impacts the peroxisome proliferator-activated receptor transcription pathway (52). Circulating levels of FABP4 are associated with endothelial dysfunction in DM patients (53) and increased risk of atherosclerosis and cerebrovascular malformations (54, 55).

Fatty acid oxidation (FAO) accounts for roughly 14% of ATP production in cultured EC (22). Carnitine palmitoyl transferase (CPT1A), a long-chain fatty acid shuttle protein regulated by AMP-activated protein kinase, is a key point of FAO regulation (22, 56, 57). Palmitate has been shown to contribute carbons to nucleotide formation *via* the tricarboxylic acid (TCA) cycle. When CPT1A was knocked down *in vitro*, vessel sprouting was impaired due to low levels of deoxy ribonucleotides. CPT1A knockdown in mice produced impaired retinal vessel formation (58).

In addition to glucose and fatty acids, amino acids contribute to EC metabolism and function (59). Specifically glutamine fuels anaplerotic reactions *via* the TCA cycle (23, 29, 60). Internalization of glutamine occurs *via* solute carrier family 1 member 5 (23, 29), and inhibition of glutaminase causes premature senescence and reduced proliferation in ECs (61). The most intensely investigated amino acid with respect to endothelial dysfunction is, however, arginine in the context of its conversion to the vasorelaxant nitric oxide (NO) by endothelial nitric oxide synthase (eNOS).

### Endothelial Nitric Oxide Is Important to Vascular Function and Its Production Is Affected by Genetic and Metabolic Factors

In addition to causing vasorelaxation, NO affects smooth muscle cell proliferation, aggregation and adhesion of platelets and leukocytes, important processes to atherosclerosis and other CVD (62, 63). When eNOS has insufficient arginine, a result of competition with arginase, and/or lacks the cofactor tetrahydrobiopterin, it produces reactive oxygen species (ROS) instead of the products NO and citrulline – in a pathological state known as uncoupling (64–71). Furthermore, the pressure of ${\text{O}}_{2}^{-}$ causes rapid inactivation of endothelium-derived NO (72). Indeed, arginase and eNOS activities and genotypes in addition to tetrahydrobiopterin levels have all been linked to endothelial function (73–76).

Altered NOS activity due to inhibition by asymmetric dimethylarginine (ADMA) encourages NOS uncoupling leading to endothelial dysfunction. ADMA levels, and the ratio of ADMA to arginine, have been connected to several aspects of CVD risk (77–80).

Genetic variation in eNOS affects some measures of recovery of blood flow control in acute myocardial infarction (73). Inhibiting arginase activity, which reduces eNOS uncoupling, is helpful in restoring endothelial function in both coronary artery disease and after ischemia–reperfusion injury (64, 65). Genetic variation in NOS1 has also been linked with CVD in various studies (75, 76). Furthermore, the ROS scavenger methionine sulfoxide reductase A, important to reducing the effect of uncoupled NOS and other ROS, is affected by genetic variation relevant to coronary artery disease risk (81, 82).

Interestingly, the extracellular presence of certain amino acids – ornithine, l-lysine, l-homoarginine, l-glutamine, l-leucine, or l-serine – decreases NO and increases endothelium-dependent vascular resistance. This effect is reversible by adding arginine to the medium and was shown to be dependent on y^+^L and y^+^ family amino acid transporters (83).

## Decoding Endothelial Metabolism and Function through Computational Modeling

The previous section highlights the complexity of the contribution of metabolism to endothelial dysfunction. Importantly, some of the most common human metabolic gene alterations impact enzymes that are of importance to endothelial metabolic phenotypes. These include pyruvate kinase and (84) glucose-6-phosphate dehydrogenase, which alters CVD risk (85), in addition to those already mentioned above. The variability of the effect of these mutations on cardiovascular phenotypes highlights the problem of untangling complex genetic diseases (12). This complexity is aggravated by lifestyle choices that impact the expression and activity of these genes (9, 86–89). How altered gene expression and the environment combine to advance CVD can, however, be explored on the metabolic level, through metabolic systems analysis using genome-scale models of endothelial metabolism. For CVD research, genome-scale modeling promises to contribute to the definition of endothelial metabolism under different physiological conditions, allow the differentiation of individual endothelial metabolic phenotypes that can be related to CVD states and ultimately contribute to individualized therapy. In the following sections, we explain the concept of GEMs, their current and potential applications toward increasing the understanding of endothelial metabolism, and how this could lead to novel discoveries to combat CVD on the individual level.

### GEMs Provide Snapshots of Metabolism

Genome-scale metabolic models are computational models that can be used to describe and investigate the metabolic flux phenotype of a cell based on disparate biochemical information. GEMs are built from biochemical component knowledgebases, also termed biochemical network reconstructions (90). Reconstructions are organism specific and account for genetic, and biochemical components, and their interactions, based on annotated biological information sourced from literature. All metabolic reactions and metabolites contained within a reconstruction can be represented as a numerical matrix, which is comprised of the stoichiometric factors of reactants and products of each metabolic reaction. In this format, the metabolome is subject to computational research allowing metabolic reaction flux at steady state through metabolic pathways to be computed (91).

Genome-scale metabolic reconstructions aim to account for as many as possible biochemical interactions that have been described in an organism (e.g., a human). While reconstructions afford a mechanistic description of genotype–phenotype relationships, they are not context specific. However, when constrained with cell or context-specific data, for example gene expression information of ECs, reconstructions afford GEMs that are descriptive of the biological event and cell of interest. Gene expression data of a HUVEC cells at normoxia vs. hypoxia would for instance generate two GEMs based on the same reconstruction thereby providing two snapshots descriptive of metabolic flux through reactions as defined by the two expression datasets. Essentially, reconstructions define the biochemical components of an organism, while context-specific polyomic data are required to generate a GEM of a particular cell or cellular event. Genomic, proteomic, and/or metabolomic fingerprints can thus be analyzed and compared within the context of GEMs (92).

The methodology of building, curating, and analyzing reconstructions and GEMs is commonly referred to as constraint-based analysis. Various software has been developed to facilitate constraint-based analysis including the COBRA and RAVEN toolbox’s for Matlab, Merlin and CORDA (93–96). Detailed protocols describing the necessary stages of building and curation are established (90, 97–99). Ultimately, constraint-based analysis of GEMs allows holistic exploration of metabolic phenotypes *in silico* and affords realistic hypotheses of biochemical mechanisms (92). In the past 5 years, multiple applications of GEMs descriptive of human metabolism have materialized that may contribute to the understanding of how genetic and environmental factors collectively contribute to CVD disease phenotypes when applied to endothelial metabolic research.

### GEMs Differentiate between Metabolic Phenotypes

In the context of CVD, GEMs that are descriptive of healthy and CVD endothelial metabolism can be produced. As recently reviewed in Väremo et al. (100), GEMs of various tissues have been built and applied to the investigation of CVD-related disorders, including DM and metabolic syndrome, although not yet endothelium (101–104). Transcriptional changes in cardiomyocytes of DM patients have been analyzed using the myocyte-specific GEM, iMyocyte2419, revealing deregulation of metabolic pathways ultimately linked to dihydro-lipoamide dehydrogenase, a unique characteristic of myocyte response in DM (101).

Genome-scale metabolic models serve as a biomarker discovery tool, and a tool to discover potentially “druggable” metabolic (105). Computational techniques exist that predict the pathways likely to be responsible for differences between two metabolic states, identifying these differences allows reactions, linked to genes in a GEM, to be selected as drug targets, for example in hepatocellular carcinoma and Alzheimer’s, or metabolites to be identified as potential biomarkers for example for drug resistance in ovarian cancer (100, 106–108). Changes due in FAO in ECs leading to alterations in EC permeability – clinically important to sepsis – have been detected using a GEM. Altering FAO using drugs was shown to alter permeability, which may be clinically useful (109), future discoveries of this type may be linked to NO synthesis or clotting factor production useful for modulating CVD risk factors.

### GEMs Can Define Endothelial Metabolism

Genome-scale metabolic models that are descriptive of core endothelial metabolism have already been produced. Patella et al recently used endothelial proteomic data to constrain the human reconstruction, Recon 1 (110), to generate a GEM that describes EC cell core metabolism during tube formation in matrigel (109). FAO was identified as an area of metabolism that is altered during tube formation. CPT1A inhibition affects ATP production *via* the TCA cycle and oxidative phosphorylation. Downstream, this alters Ca^2+^ signaling and junctional proteins *via* phospho-signaling to alter endothelial permeability, which were partially reversed by pyruvate supplementation (109). Automated GEMs have also been generated for colon and cerebral cortex ECs (111), though these models were not applied to CVD research.

Although automatically generated GEMs of EC metabolism have been used to reveal basal endothelial metabolic pathway usage, further curation and validation of EC GEMs would be beneficial. Investigations of vascular endothelial metabolism in different conditions and with different genetic backgrounds could be achieved, allowing genetic variation outside the context of core energy metabolism to be queried. For example, due to the inherent connectivity of metabolic reactions within GEMs, alterations in the release of sphingosine-1-P (a sphingolipid involved in vascular and immune signaling pathways) from ECs could be hypothesized and related to alterations in core energy metabolism induced by global metabolic expression profiles. The release of sphingosine-1-P from ECs and its contribution to individual vascular health could thus be proposed on biochemical alterations on the systems level as opposed to mutations in sphingosine kinase alone.

### GEMs Can Be Personalized to Account for Individual Genetic Variation

Computational modeling can contribute to decisions regarding the suitability of a treatment for individual patients. GEMs could be produced for individuals based on genomics and subsequently used to stratify patients and personalize medical interventions for CVD. GEMs maybe based on generalized transcriptomic data from a pool of samples from a cell type (112) or a set of models may be created from individual samples and comparing the metabolic phenotypes predicted by each, allowing links between metabolism and broader phenotype, such as drug resistance in cancer cells, to be explored and may lead to insights about predictive biomarkers and druggable targets (108, 113, 114). Various algorithms for selecting active reactions for context-specific models based on transcriptomic and proteomic data are available including INIT and iMAT. These approaches have differing strengths and weaknesses that have been described and compared elsewhere (98).

**Figure 2:**
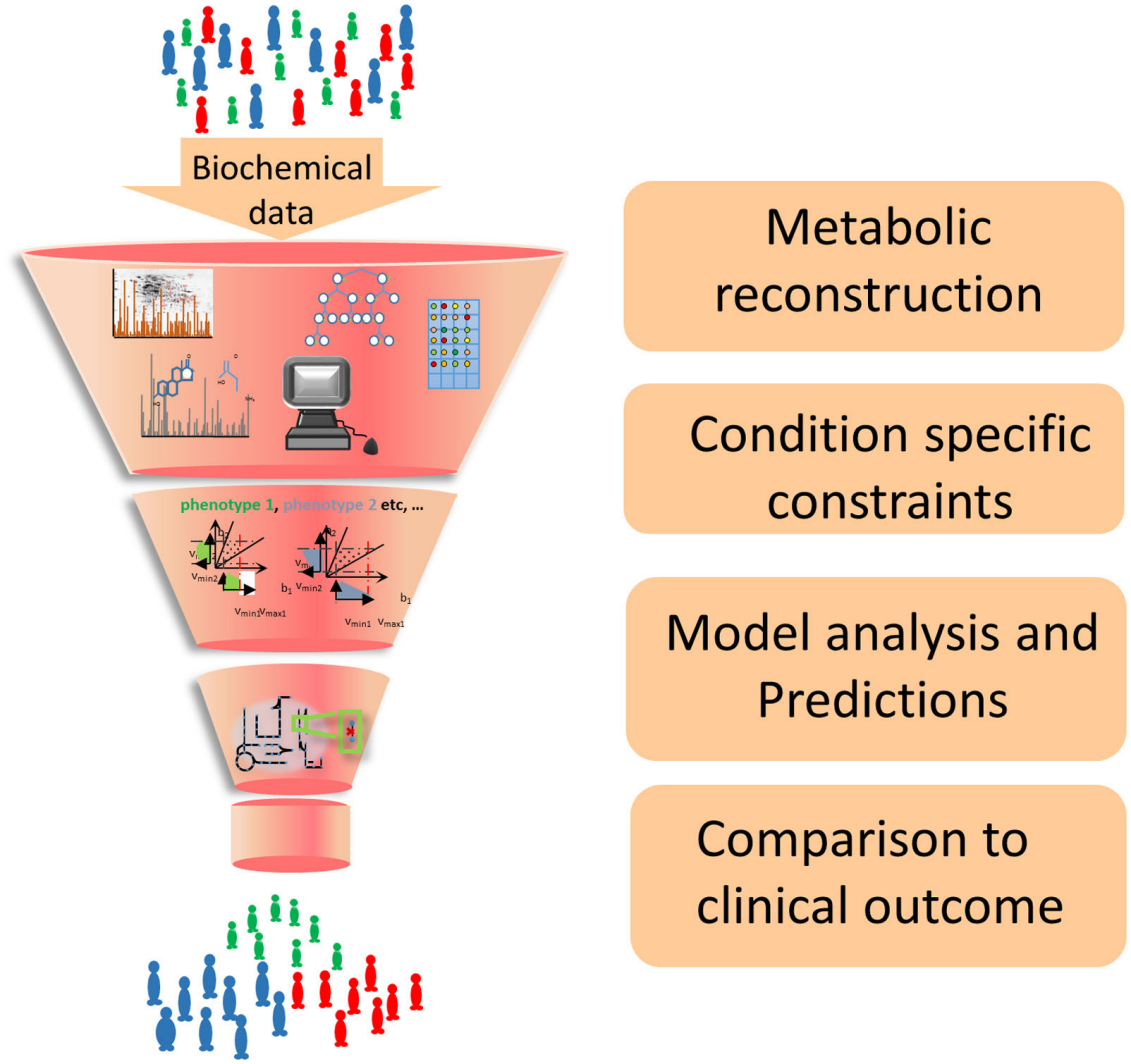
**Workflow of GEM construction and contribution to developing new strategies for the clinic**. Biochemical data from cell culture and clinical studies are combined to form a comprehensive metabolic reconstruction, which is constrained to form a context-specific GEM and produce biologically well-founded predictions that will suggest future clinical interventions.

Individualized hepatocellular carcinoma models have been used to predict patient outcomes based on the predicted production of acetate, identified as a key metabolic pathway for survival (114). Twenty-four individualized GEMs of erythrocytes were created based on genetic and metabolic data. These captured altered dynamics of erythrocyte metabolism and allowed the identification of individuals at risk to drug-induced anemia based upon their genomic sequence (115). These examples highlight a potential workflow, exemplified in Figure 2, to contribute to the personalization and stratification of medical treatments in the clinic. In the future, it is envisioned that an EC GEM could be used in a similar fashion by comparing GEMs CVD patients and healthy individuals to identify key metabolic changes to CVD for example those that increase production of atherosclerotic plaques.

## Conclusion

Developing personalized CVD therapeutic interventions relies on the ability to account for genotypic and phenotypic variation. Variability in disease phenotypes can be captured and understood in the context of GEM’s to facilitate this process.

Genome-scale metabolic models provide an integrated approach in studying EC metabolism. They allow analysis of the multiple factors affecting ECs in the body, facilitating the exploration of the relationship of genotype to metabolic phenotype. This offers the possibility of producing personalized predictions of CVD risk and treatment, that account for both genetic and lifestyle factors. Currently, GEMs are the only biochemical model type that can account for both of these factors within a predictive modeling framework (92).

Genome-scale metabolic models are only one type of model used to account for EC function. Focused and mechanistic computational models of various aspects of vascular biology have also been made. These address some important biophysical parameters that are currently outside the scope of GEM modeling. This includes assessing the effects of shear stress on blood vessel reactivity and growth as well as the effects on blood cell/endothelium interactions of flow (116–122). Models describing the effects of circulation on endothelial metabolites have also been built (123). Endothelial NO interactions (124–126), Ca^2+^ signaling (127) along with protein (128) and mechanical (119) signaling have also been addressed with computational modeling. Models have been individualized using patient data and have explored the effects of stenting on blood flow (129–132).

Integrating biophysical and signaling parameters with GEMs would generate a more complete understanding of the role of endothelial metabolism for CVD. In addition, these future GEMs would allow retrospective analysis of biophysical and genomic data that have been generated in the last few decades from population studies (86, 133), whose analysis is currently confined to multivariate statistical and comparative analysis techniques for the identification of CVD risk factors. Such an effort could allow, for example, *in silico* querying of the effect of LDL deposition on global endothelial metabolism. Indeed, computational analysis of LDL metabolism has already proposed novel approaches to combat CVD (134–136).

Realistic computational predictions of the effects of genetic and environmental perturbations on endothelial metabolism are possible and beneficial. There has been some exploration of CVD with GEMs and analysis of EC metabolism with GEMs; however, the full potential of this technique is only just beginning to be explored. Existing and future models will allow clinicians and researchers to investigate variable endothelial function *in silico* in a data-driven manner, to optimize future clinical interventions.

## Author Contributions

SM and OR wrote the manuscript and conceived the ideology. HH, SP, and PJ conceived the ideology and contributed to writing of the manuscript.

## Conflict of Interest Statement

The authors declare that the research was conducted in the absence of any commercial or financial relationships that could be construed as a potential conflict of interest.

## References

[1] WHO. Cardiovascular Diseases (CVDs). World Health Organization (2016). Available from: http://www.who.int/mediacentre/factsheets/fs317/en/

[2] Field JM Hazinski MF Sayre MR Chameides L Schexnayder SM Hemphill R Part 1: executive summary: 2010 American Heart Association guidelines for cardiopulmonary resuscitation and emergency cardiovascular care. Circulation (2010) 122(Suppl 3):640–57. 10.1161/CIRCULATIONAHA.110.970889 20956217

[3] Townsend N Nichols M Scarborough P Rayner M Cardiovascular disease in Europe – epidemiological update 2015. Eur Heart J (2015) 36(40):ehv428 10.1093/eurheartj/ehv428 26306399

[4] Landmesser U Drexler H. The clinical significance of endothelial dysfunction. Curr Opin Cardiol (2005) 20(6):547–51. 10.1097/01.hco.0000179821.11071.79 16234629

[5] Vanhoutte PM Shimokawa H Feletou M Tang EHC Endothelial dysfunction and vascular disease – a thirthieth anniversary update. Acta Physiol (Oxf) (2015). 10.1111/apha.12646 26706498

[6] Kazmi R Boyce S Lwaleed B. Homeostasis of hemostasis: the role of endothelium. Semin Thromb Hemost (2015) 41(6):549–55. 10.1055/s-0035-1556586 26270112

[7] Santillo M Colantuoni A Mondola P Guida B Damiano S. NOX signaling in molecular cardiovascular mechanisms involved in the blood pressure homeostasis. Front Physiol (2015) 6:194. 10.3389/fphys.2015.00194 26217233 PMC4493385

[8] Shen X Tan Z Zhong X Tian Y Wang X Yu B Endocardial endothelium is a key determinant of force-frequency relationship in rat ventricular myocardium. J Appl Physiol (2013) 115(3):383–93. 10.1152/japplphysiol.01415.2012 23703113 PMC3743009

[9] Choi B Steiss D Garcia-Rivas J Kojaku S Schnall P Dobson M Comparison of body mass index with waist circumference and skinfold-based percent body fat in firefighters: adiposity classification and associations with cardiovascular disease risk factors. Int Arch Occup Environ Health (2016) 89(3):435–48. 10.1007/s00420-015-1082-6 26254211

[10] Hwang M-H Kim S Type 2 diabetes: endothelial dysfunction and exercise. J Exerc Nutrition Biochem (2014) 18(3):239–47. 10.5717/jenb.2014.18.3.239 PMC4241901 25566460

[11] Kwaśniewska M Kozińska J Dziankowska-Zaborszczyk E Kostka T Jegier A Rębowska E The impact of long-term changes in metabolic status on cardiovascular biomarkers and microvascular endothelial function in middle-aged men: a 25-year prospective study. Diabetol Metab Syndr (2015) 7:81. 10.1186/s13098-015-0074-8 26388952 PMC4573488

[12] Hamrefors V. Common genetic risk factors for coronary artery disease: new opportunities for prevention? Clin Physiol Funct Imaging (2015). 10.1111/cpf.12289 26278888

[13] Rolfsson O Palsson B Thiele I. The human metabolic reconstruction Recon 1 directs hypotheses of novel human metabolic functions. BMC Syst Biol (2011) 5:155. 10.1186/1752-0509-5-155 21962087 PMC3224382

[14] Parra-Bonilla G Alvarez DF Alexeyev M Vasauskas A Stevens T. Lactate dehydrogenase a expression is necessary to sustain rapid angiogenesis of pulmonary microvascular endothelium. PLoS One (2013) 8(9):e75984. 10.1371/journal.pone.0075984 24086675 PMC3784391

[15] Tuleta I França CN Wenzel D Fleischmann B Nickenig G Werner N Intermittent hypoxia impairs endothelial function in early preatherosclerosis. Adv Exp Med Biol (2015) 858:1–7. 10.1007/5584_2015_114 26017722

[16] Doddaballapur A Michalik KM Manavski Y Lucas T Houtkooper RH You X Laminar shear stress inhibits endothelial cell metabolism via KLF2-mediated repression of PFKFB3. Arterioscler Thromb Vasc Biol (2015) 35(1):137–45. 10.1161/ATVBAHA.114.304277 25359860

[17] Zhang Z Apse K Pang J Stanton RC. High glucose inhibits glucose-6-phosphate dehydrogenase via cAMP in aortic endothelial cells. J Biol Chem (2000) 275(51):40042–7. 10.1074/jbc.M007505200 11007790

[18] Halldórsson H Thors B Thorgeirsson G Thrombin or Ca(++)-ionophore-mediated fall in endothelial ATP levels independent of poly(ADP-Ribose) polymerase activity and NAD levels – comparison with the effects of hydrogen peroxide. Nucleosides Nucleotides Nucleic Acids (2015) 34(4):246–57. 10.1080/15257770.2014.984072 25774718

[19] Mahajan-Thakur S Böhm A Jedlitschky G Schrör K Rauch BH. Sphingosine-1-phosphate and its receptors: a mutual link between blood coagulation and inflammation. Mediators Inflamm (2015) 2015:831059. 10.1155/2015/831059 26604433 PMC4641948

[20] Viator RJ Khader H Hingorani N Long S Solodushko V Fouty B Hypoxia-induced increases in glucose uptake do not cause oxidative injury or advanced glycation end-product (AGE) formation in vascular endothelial cells. Physiol Rep (2015) 3(7):e12460 10.14814/phy2.12460 26177960 PMC4552536

[21] Koziel A Woyda-Ploszczyca A Kicinska A Jarmuszkiewicz W. The influence of high glucose on the aerobic metabolism of endothelial EA.hy926 cells. Pflugers Arch (2012) 464(6):657–69. 10.1007/s00424-012-1156-1 23053476 PMC3513600

[22] Dagher Z Ruderman N Tornheim K Ido Y. Acute regulation of fatty acid oxidation and AMP-activated protein kinase in human umbilical vein endothelial cells. Circ Res (2001) 88(12):1276–82. 10.1161/hh1201.092998 11420304

[23] Lohmann R Souba WW Bode BP. Rat liver endothelial cell glutamine transporter and glutaminase expression contrast with parenchymal cells. Am J Physiol (1999) 276(3 Pt 1):G743–50. 10070052 10.1152/ajpgi.1999.276.3.G743

[24] Eelen G de Zeeuw P Simons M Carmeliet P. Endothelial cell metabolism in normal and diseased vasculature. Circ Res (2015) 116(7):1231–44. 10.1161/CIRCRESAHA.116.302855 25814684 PMC4380230

[25] Tabas I García-Cardeña G Owens G. Recent insights into the cellular biology of atherosclerosis. J Cell Biol (2015) 209(1):13–22. 10.1083/jcb.201412052 25869663 PMC4395483

[26] De Bock K Georgiadou M Schoors S Kuchnio A Wong B Cantelmo A Role of PFKFB3-driven glycolysis in vessel sprouting. Cell (2013) 154(3):651–63. 10.1016/j.cell.2013.06.037 23911327

[27] Dobrina A Rossi F. Metabolic properties of freshly isolated bovine endothelial cells. Biochim Biophys Acta (1983) 762(2):295–301. 10.1016/0167-4889(83)90084-8 6830877

[28] Krützfeldt A Spahr R Mertens S Siegmund B Piper HM. Metabolism of exogenous substrates by coronary endothelial cells in culture. J Mol Cell Cardiol (1990) 22(12):1393–404. 10.1016/0022-2828(90)90984-A 2089157

[29] Leighton B Curi R Hussein A Newsholme EA. Maximum activities of some key enzymes of glycolysis, glutaminolysis, Krebs cycle and fatty acid utilization in bovine pulmonary endothelial cells. FEBS Lett (1987) 225(1–2):93–6. 10.1016/0014-5793(87)81137-7 3691808

[30] Parmar KM Larman HB Dai G Zhang Y Wang ET Moorthy SN Integration of flow-dependent endothelial phenotypes by Kruppel-like factor 2. J Clin Invest (2006) 116(1):49–58. 10.1172/JCI24787 16341264 PMC1307560

[31] Xu Y An X Guo X Habtetsion TG Wang Y Xu X Endothelial PFKFB3 plays a critical role in angiogenesis. Arterioscler Thromb Vasc Biol (2014) 34(6):1231–9. 10.1161/ATVBAHA.113.303041 24700124 PMC4120754

[32] Zhang X-G Zhang Y-Q Zhao D-K Wu J-X Zhao J Jiao X-M Relationship between blood glucose fluctuation and macrovascular endothelial dysfunction in type 2 diabetic patients with coronary heart disease. Eur Rev Med Pharmacol Sci (2014) 18(23):3593–600. 25535128

[33] Temelkova-Kurktschiev TS Koehler C Henkel E Leonhardt W Fuecker K Hanefeld M. Postchallenge plasma glucose and glycemic spikes are more strongly associated with atherosclerosis than fasting glucose or HbA1c level. Diabetes Care (2000) 23(12):1830–4. 10.2337/diacare.23.12.1830 11128361

[34] Azuma K Kawamori R Toyofuku Y Kitahara Y Sato F Shimizu T Repetitive fluctuations in blood glucose enhance monocyte adhesion to the endothelium of rat thoracic aorta. Arterioscler Thromb Vasc Biol (2006) 26(10):2275–80. 10.1161/01.ATV.0000239488.05069.03 16888238

[35] Torimoto K Okada Y Mori H Tanaka Y. Relationship between fluctuations in glucose levels measured by continuous glucose monitoring and vascular endothelial dysfunction in type 2 diabetes mellitus. Cardiovasc Diabetol (2013) 12:1. 10.1186/1475-2840-12-1 23280391 PMC3557219

[36] Jiao X-M Zhang X-G Xu XU-P Yi C Bin C Cheng Q-P Blood glucose fluctuation aggravates lower extremity vascular disease in type 2 diabetes. Eur Rev Med Pharmacol Sci (2014) 18(14):2025–30. 25027342

[37] Service FJ Molnar GD Rosevear JW Ackerman E Gatewood LC Taylor WF Mean amplitude of glycemic excursions, a measure of diabetic instability. Diabetes (1970) 19(9):644–55. 10.2337/diab.19.9.644 5469118

[38] Du X Matsumura T Edelstein D Rossetti L Zsengellér Z Szabó C Inhibition of GAPDH activity by poly(ADP-ribose) polymerase activates three major pathways of hyperglycemic damage in endothelial cells. J Clin Invest (2003) 112(7):1049–57. 10.1172/JCI18127 14523042 PMC198524

[39] Kim DH Seok YM Kim IK Lee I-K Jeong SY Jeoung NH. Glucosamine increases vascular contraction through activation of RhoA/Rho kinase pathway in isolated rat aorta. BMB Rep (2011) 44(6):415–20. 10.5483/BMBRep.2011.44.6.415 21699756

[40] Rajapakse AG Ming X-F Carvas JM Yang Z. O-linked beta-N-acetylglucosamine during hyperglycemia exerts both anti-inflammatory and pro-oxidative properties in the endothelial system. Oxid Med Cell Longev (2009) 2(3):172–5. 10.4161/oxim.2.3.8482 20592773 PMC2763244

[41] Rajapakse AG Ming X-F Carvas JM Yang Z. The hexosamine biosynthesis inhibitor azaserine prevents endothelial inflammation and dysfunction under hyperglycemic condition through antioxidant effects. Am J Physiol Heart Circ Physiol (2009) 296(3):H815–22. 10.1152/ajpheart.00756.2008 19136606

[42] Wu G Haynes TE Yan W Meininger CJ. Presence of glutamine:fructose-6-phosphate amidotransferase for glucosamine-6-phosphate synthesis in endothelial cells: effects of hyperglycaemia and glutamine. Diabetologia (2001) 44(2):196–202. 10.1007/s001250051599 11270676

[43] Slawson C Copeland RJ Hart GW. O-GlcNAc signaling: a metabolic link between diabetes and cancer? Trends Biochem Sci (2010) 35(10):547–55. 10.1016/j.tibs.2010.04.005 20466550 PMC2949529

[44] Benedito R Roca C Sörensen I Adams S Gossler A Fruttiger M The notch ligands Dll4 and Jagged1 have opposing effects on angiogenesis. Cell (2009) 137(6):1124–35. 10.1016/j.cell.2009.03.025 19524514

[45] Vaisman N Gospodarowicz D Neufeld G. Characterization of the receptors for vascular endothelial growth factor. J Biol Chem (1990) 265(32):19461–6. 2246236

[46] Federici M Menghini R Mauriello A Hribal ML Ferrelli F Lauro D Insulin-dependent activation of endothelial nitric oxide synthase is impaired by O-linked glycosylation modification of signaling proteins in human coronary endothelial cells. Circulation (2002) 106(4):466–72. 10.1161/01.CIR.0000023043.02648.51 12135947

[47] Du XL Edelstein D Dimmeler S Ju Q Sui C Brownlee M. Hyperglycemia inhibits endothelial nitric oxide synthase activity by posttranslational modification at the Akt site. J Clin Invest (2001) 108(9):1341–8. 10.1172/JCI11235 11696579 PMC209429

[48] Wautier J-L Schmidt AM. Protein glycation: a firm link to endothelial cell dysfunction. Circ Res (2004) 95(3):233–8. 10.1161/01.RES.0000137876.28454.64 15297385

[49] Wautier J-L Wautier M-P. Erythrocytes and platelet adhesion to endothelium are mediated by specialized molecules. Clin Hemorheol Microcirc (2004) 30(3–4):181–4. 15258342

[50] Vlassara H Fuh H Donnelly T Cybulsky M. Advanced glycation endproducts promote adhesion molecule (VCAM-1, ICAM-1) expression and atheroma formation in normal rabbits. Mol Med (1995) 1(4):447–56. 8521302 PMC2229997

[51] Kajikawa M Nakashima A Fujimura N Maruhashi T Iwamoto Y Iwamoto A Ratio of serum levels of AGEs to soluble form of RAGE is a predictor of endothelial function. Diabetes Care (2015) 38(1):119–25. 10.2337/dc14-1435 25336748

[52] Makowski L Brittingham KC Reynolds JM Suttles J Hotamisligil GS. The fatty acid-binding protein, aP2, coordinates macrophage cholesterol trafficking and inflammatory activity. Macrophage expression of aP2 impacts peroxisome proliferator-activated receptor gamma and IkappaB kinase activities. J Biol Chem (2005) 280(13):12888–95. 10.1074/jbc.M413788200 15684432 PMC3493120

[53] Aragonès G Ferré R Lázaro I Cabré A Plana N Merino J Fatty acid-binding protein 4 is associated with endothelial dysfunction in patients with type 2 diabetes. Atherosclerosis (2010) 213(1):329–31. 10.1016/j.atherosclerosis.2010.07.026 20723896

[54] Yeung DCY Xu A Cheung CWS Wat NMS Yau MH Fong CHY Serum adipocyte fatty acid-binding protein levels were independently associated with carotid atherosclerosis. Arterioscler Thromb Vasc Biol (2007) 27(8):1796–802. 10.1161/ATVBAHA.107.146274 17510463

[55] Cataltepe S Arikan MC Liang X Smith TW Cataltepe O. Fatty acid binding protein 4 expression in cerebral vascular malformations: implications for vascular remodelling. Neuropathol Appl Neurobiol (2015) 41(5):646–56. 10.1111/nan.12159 24865546

[56] Dagher Z Ruderman N Tornheim K Ido Y. The effect of AMP-activated protein kinase and its activator AICAR on the metabolism of human umbilical vein endothelial cells. Biochem Biophys Res Commun (1999) 265(1):112–5. 10.1006/bbrc.1999.1635 10548499

[57] Zabielska MA Borkowski T Slominska EM Smolenski RT. Inhibition of AMP deaminase as therapeutic target in cardiovascular pathology. Pharmacol Rep (2015) 67(4):682–8. 10.1016/j.pharep.2015.04.007 26321268

[58] Schoors S Bruning U Missiaen R Queiroz KCS Borgers G Elia I Fatty acid carbon is essential for dNTP synthesis in endothelial cells. Nature (2015) 520(7546):192–7. 10.1038/nature14362 25830893 PMC4413024

[59] Pan M Fischer CP Wasa M Lukaszewicz G Stevens BR Bode BP Amino acid metabolism and the vascular endothelium: regulation and disease implications. Shock (1995) 4(2):79–88. 10.1097/00024382-199508000-00001 7496902

[60] Wu G Haynes TE Li H Meininger CJ. Glutamine metabolism in endothelial cells: ornithine synthesis from glutamine via pyrroline-5-carboxylate synthase. Comp Biochem Physiol Part A Mol Integr Physiol (2000) 126(1):115–23. 10.1016/S1095-6433(00)00196-3 10908859

[61] Unterluggauer H Mazurek S Lener B Hütter E Eigenbrodt E Zwerschke W Premature senescence of human endothelial cells induced by inhibition of glutaminase. Biogerontology (2008) 9(4):247–59. 10.1007/s10522-008-9134-x 18317946

[62] Kawashima S Yokoyama M. Dysfunction of endothelial nitric oxide synthase and atherosclerosis. Arterioscler Thromb Vasc Biol (2004) 24(6):998–1005. 10.1161/01.ATV.0000125114.88079.96 15001455

[63] Yang Y Wu Z Meininger CJ Wu G. l-Leucine and NO-mediated cardiovascular function. Amino Acids (2015) 47(3):435–47. 10.1007/s00726-014-1904-y 25552397

[64] Kovamees O Shemyakin A Eriksson M Angelin B Pernow J. Arginase inhibition improves endothelial function in patients with familial hypercholesterolaemia irrespective of their cholesterol levels. J Intern Med (2015). 10.1111/joim.12461 26707366

[65] Kövamees O Shemyakin A Pernow J. Effect of arginase inhibition on ischemia-reperfusion injury in patients with coronary artery disease with and without diabetes mellitus. PLoS One (2014) 9(7):e103260. 10.1371/journal.pone.0103260 25072937 PMC4114552

[66] Daff S. NO synthase: structures and mechanisms. Nitric Oxide (2010) 23(1):1–11. 10.1016/j.niox.2010.03.001 20303412

[67] Vásquez-Vivar J Kalyanaraman B Martásek P Hogg N Masters BS Karoui H Superoxide generation by endothelial nitric oxide synthase: the influence of cofactors. Proc Natl Acad Sci U S A (1998) 95(16):9220–5. 10.1073/pnas.95.16.9220 9689061 PMC21319

[68] Vásquez-Vivar J Martásek P Whitsett J Joseph J Kalyanaraman B. The ratio between tetrahydrobiopterin and oxidized tetrahydrobiopterin analogues controls superoxide release from endothelial nitric oxide synthase: an EPR spin trapping study. Biochem J (2002) 362(Pt 3):733–9. 10.1042/bj3620733 11879202 PMC1222439

[69] Ali ZA Rinze R Douglas G Hu Y Xiao Q Qi W Tetrahydrobiopterin determines vascular remodeling through enhanced endothelial cell survival and regeneration. Circulation (2013) 128(11 Suppl 1):S50–8. 10.1161/CIRCULATIONAHA.112.000249 24030421 PMC5357046

[70] Xia Y Tsai AL Berka V Zweier JL. Superoxide generation from endothelial nitric-oxide synthase. A Ca2+/calmodulin-dependent and tetrahydrobiopterin regulatory process. J Biol Chem (1998) 273(40):25804–8. 10.1074/jbc.273.40.25804 9748253

[71] Landmesser U Dikalov S Price SR McCann L Fukai T Holland SM Oxidation of tetrahydrobiopterin leads to uncoupling of endothelial cell nitric oxide synthase in hypertension. J Clin Invest (2003) 111(8):1201–9. 10.1172/JCI200314172 12697739 PMC152929

[72] Paolocci N Biondi R Bettini M Lee C-I Berlowitz CO Rossi R Oxygen radical-mediated reduction in basal and agonist-evoked NO release in isolated rat heart. J Mol Cell Cardiol (2001) 33(4):671–9. 10.1006/jmcc.2000.1334 11341236

[73] Machado-Silva W Alfinito-Kreis R Carvalho LSF Quinaglia-E-Silva JC Almeida OLR Brito CJ Endothelial nitric oxide synthase genotypes modulate peripheral vasodilatory properties after myocardial infarction. Gene (2015) 568(2):165–9. 10.1016/j.gene.2015.05.042 26002446

[74] Umman B Cakmakoglu B Cincin ZB Kocaaga M Emet S Tamer S Identification of gene variants related to the nitric oxide pathway in patients with acute coronary syndrome. Gene (2015) 574(1):76–81. 10.1016/j.gene.2015.07.081 26232608

[75] Levinsson A Olin A-C Björck L Rosengren A Nyberg F. Nitric oxide synthase (NOS) single nucleotide polymorphisms are associated with coronary heart disease and hypertension in the INTERGENE study. Nitric Oxide (2014) 39:1–7. 10.1016/j.niox.2014.03.164 24713495

[76] Manso H Krug T Sobral J Albergaria I Gaspar G Ferro JM Variants within the nitric oxide synthase 1 gene are associated with stroke susceptibility. Atherosclerosis (2012) 220(2):443–8. 10.1016/j.atherosclerosis.2011.11.011 22153699

[77] Stamboul K Lorin J Lorgis L Guenancia C Beer J-C Touzery C Atrial fibrillation is associated with a marker of endothelial function and oxidative stress in patients with acute myocardial infarction. PLoS One (2015) 10(7):e0131439. 10.1371/journal.pone.0131439 26158510 PMC4497674

[78] Xuan C Tian Q-W Li H Zhang B-B He G-W Lun L-M Levels of asymmetric dimethylarginine (ADMA), an endogenous nitric oxide synthase inhibitor, and risk of coronary artery disease: a meta-analysis based on 4713 participants. Eur J Prev Cardiol (2016) 23(5):502–10. 10.1177/2047487315586094 25956428

[79] Shivkar RR Abhang SA. Ratio of serum asymmetric dimethyl arginine (ADMA)/nitric oxide in coronary artery disease patients. J Clin Diagn Res (2014) 8(8):CC04–6. 10.7860/JCDR/2014/7849.4665 25302189 PMC4190709

[80] Masaki N Hakuno D Toya T Shiraishi Y Kujiraoka T Namba T Association between brachial-ankle pulse wave velocity and the ratio of l-arginine to asymmetric dimethylarginine in patients undergoing coronary angiography. J Cardiol (2015) 65(4):311–7. 10.1016/j.jjcc.2014.06.005 25043133

[81] Tabas I Garcia-Cardena G Owens GK The cell biology of disease: recent insights into the cellular biology of atherosclerosis. J Cell Biol (2015) 209(1):13–22. 10.1083/jcb.201412052 25869663 PMC4395483

[82] García-Bermúdez M López-Mejías R González-Juanatey C Castañeda S Miranda-Filloy JA Blanco R Association of the methionine sulfoxide reductase A rs10903323 gene polymorphism with cardiovascular disease in patients with rheumatoid arthritis. Scand J Rheumatol (2012) 41(5):350–3. 10.3109/03009742.2012.677063 22657383

[83] Kakoki M Kim H-S Edgell C-JS Maeda N Smithies O Mattson DL. Amino acids as modulators of endothelium-derived nitric oxide. Am J Physiol Renal Physiol (2006) 291(2):F297–304. 10.1152/ajprenal.00417.2005 16571593

[84] Carey PJ Chandler J Hendrick A Reid MM Saunders PW Tinegate H Prevalence of pyruvate kinase deficiency in northern European population in the north of England. Northern Region Haematologists Group. Blood (2000) 96(12):4005–6. 11186276

[85] Meloni L Manca MR Loddo I Cioglia G Cocco P Schwartz A Glucose-6-phosphate dehydrogenase deficiency protects against coronary heart disease. J Inherit Metab Dis (2008) 31(3):412–7. 10.1007/s10545-008-0704-5 18392752

[86] Tsao CW Vasan RS. Cohort profile: the Framingham Heart Study (FHS): overview of milestones in cardiovascular epidemiology. Int J Epidemiol (2015) 44(6):1800–13. 10.1093/ije/dyv337 26705418 PMC5156338

[87] Jankovic N Geelen A Streppel MT de Groot LC Kiefte-de Jong JC Orfanos P WHO guidelines for a healthy diet and mortality from cardiovascular disease in European and American elderly: the CHANCES project. Am J Clin Nutr (2015) 102(4):745–56. 10.3945/ajcn.114.095117 26354545 PMC4588736

[88] Miao C Bao M Xing A Chen S Wu Y Cai J Cardiovascular health score and the risk of cardiovascular diseases. PLoS One (2015) 10(7):e0131537. 10.1371/journal.pone.0131537 26154254 PMC4495991

[89] Eckel N Meidtner K Kalle-Uhlmann T Stefan N Schulze MB. Metabolically healthy obesity and cardiovascular events: a systematic review and meta-­analysis. Eur J Prev Cardiol (2015) 1–11. 10.1177/2047487315623884 26701871

[90] Thiele I Palsson B. A protocol for generating a high-quality genome-scale metabolic reconstruction. Nat Protoc (2010) 5(1):93–121. 10.1038/nprot.2009.203 20057383 PMC3125167

[91] Rolfsson Ó Palsson BO Decoding the jargon of bottom-up metabolic systems biology. Bioessays (2015) 37(6):588–91. 10.1002/bies.201400187 25761171

[92] Bordbar A Monk J King Z Palsson B. Constraint-based models predict metabolic and associated cellular functions. Nat Rev Genet (2014) 15(2):107–20. 10.1038/nrg3643 24430943

[93] Becker S Feist A Mo M Hannum G Palsson B Herrgard M. Quantitative prediction of cellular metabolism with constraint-based models: the COBRA toolbox. Nat Protoc (2007) 2(3):727–38. 10.1038/nprot.2007.99 17406635

[94] Agren R Liu L Shoaie S Vongsangnak W Nookaew I Nielsen J. The RAVEN toolbox and its use for generating a genome-scale metabolic model for *Penicillium chrysogenum*. PLoS Comput Biol (2013) 9(3):e1002980. 10.1371/journal.pcbi.1002980 23555215 PMC3605104

[95] Dias O Rocha M Ferreira EC Rocha I. Reconstructing genome-scale metabolic models with merlin. Nucleic Acids Res (2015) 43(8):3899–910. 10.1093/nar/gkv294 25845595 PMC4417185

[96] Schultz A Qutub AA. Reconstruction of tissue-specific metabolic networks using CORDA. PLoS Comput Biol (2016) 12(3):e1004808. 10.1371/journal.pcbi.1004808 26942765 PMC4778931

[97] Pfau T Pacheco MP Sauter T. Towards improved genome-scale metabolic network reconstructions: unification, transcript specificity and beyond. Brief Bioinform (2015):bbv100. 10.1093/bib/bbv100 26615025 PMC5142010

[98] Machado D Herrgård M. Systematic evaluation of methods for integration of transcriptomic data into constraint-based models of metabolism. PLoS Comput Biol (2014) 10(4):e1003580. 10.1371/journal.pcbi.1003580 24762745 PMC3998872

[99] Mardinoglu A Gatto F Nielsen J. Genome-scale modeling of human metabolism – a systems biology approach. Biotechnol J (2013) 8(9):985–96. 10.1002/biot.201200275 23613448

[100] Väremo L Nookaew I Nielsen J. Novel insights into obesity and diabetes through genome-scale metabolic modeling. Front Physiol (2013) 4:92. 10.3389/fphys.2013.00092 23630502 PMC3635026

[101] Väremo L Scheele C Broholm C Mardinoglu A Kampf C Asplund A Proteome- and transcriptome-driven reconstruction of the human myocyte metabolic network and its use for identification of markers for diabetes. Cell Rep (2015) 11(6):921–33. 10.1016/j.celrep.2015.04.010 25937284

[102] Kumar A Harrelson T Lewis NE Gallagher EJ LeRoith D Shiloach J Multi-tissue computational modeling analyzes pathophysiology of type 2 diabetes in MKR mice. PLoS One (2014) 9(7):e102319. 10.1371/journal.pone.0102319 25029527 PMC4100879

[103] Mardinoglu A Kampf C Asplund A Fagerberg L Hallström BM Edlund K Defining the human adipose tissue proteome to reveal metabolic alterations in obesity. J Proteome Res (2014) 13(11):5106–19. 10.1021/pr500586e 25219818

[104] Edwards LM Sigurdsson MI Robbins PA Weale ME Cavalleri GL Montgomery HE Genome-scale methods converge on key mitochondrial genes for the survival of human cardiomyocytes in hypoxia. Circ Cardiovasc Genet (2014) 7(4):407–15. 10.1161/CIRCGENETICS.113.000269 24873932

[105] Yizhak K Gabay O Cohen H Ruppin E. Model-based identification of drug targets that revert disrupted metabolism and its application to ageing. Nat Commun (2013) 4:2632. 10.1038/ncomms3632 24153335

[106] Stempler S Yizhak K Ruppin E Integrating transcriptomics with metabolic modeling predicts biomarkers and drug targets for Alzheimer’s disease. PLoS One (2014) 9(8):e105383 10.1371/journal.pone.0105383 25127241 PMC4134302

[107] Shlomi T Cabili MN Ruppin E. Predicting metabolic biomarkers of human inborn errors of metabolism. Mol Syst Biol (2009) 5(1):263. 10.1038/msb.2009.22 19401675 PMC2683725

[108] Agren R Mardinoglu A Asplund A Kampf C Uhlen M Nielsen J. Identification of anticancer drugs for hepatocellular carcinoma through personalized genome-scale metabolic modeling. Mol Syst Biol (2014) 10(3):721. 10.1002/msb.145122 24646661 PMC4017677

[109] Patella F Schug Z Persi E Neilson L Erami Z Avanzato D Proteomics-based metabolic modeling reveals that fatty acid oxidation (FAO) controls endothelial cell (EC) permeability. Mol Cell Proteomics (2015) 14(3):621–34. 10.1074/mcp.M114.045575 25573745 PMC4349982

[110] Duarte N Becker S Jamshidi N Thiele I Mo M Vo T Global reconstruction of the human metabolic network based on genomic and bibliomic data. Proc Natl Acad Sci U S A (2007) 104(6):1777–82. 10.1073/pnas.0610772104 17267599 PMC1794290

[111] Agren R Bordel S Mardinoglu A Pornputtapong N Nookaew I Nielsen J. Reconstruction of genome-scale active metabolic networks for 69 human cell types and 16 cancer types using INIT. PLoS Comput Biol (2012) 8(5):e1002518. 10.1371/journal.pcbi.1002518 22615553 PMC3355067

[112] Fouladiha H Marashi S-A Shokrgozar M. Reconstruction and validation of a constraint-based metabolic network model for bone marrow-derived mesenchymal stem cells. Cell Prolif (2015) 48(4):475–85. 10.1111/cpr.12197 26132591 PMC6496242

[113] Motamedian E Ghavami G Sardari S. Investigation on metabolism of cisplatin resistant ovarian cancer using a genome scale metabolic model and microarray data. Iran J Basic Med Sci (2015) 18(3):267–76. 25945240 PMC4414993

[114] Björnson E Mukhopadhyay B Asplund A Pristovsek N Cinar R Romeo S Stratification of hepatocellular carcinoma patients based on acetate utilization. Cell Rep (2015) 13(9):2014–26. 10.1016/j.celrep.2015.10.045 26655911

[115] Bordbar A McCloskey D Zielinski DC Sonnenschein N Jamshidi N Palsson BO Personalized whole-cell kinetic models of metabolism for discovery in genomics and pharmacodynamics. Cell Syst (2015) 1(4):283–92. 10.1016/j.cels.2015.10.003 27136057

[116] Kirby P Buerk DG Parikh J Barbee KA Jaron D Mathematical model for shear stress dependent NO and adenine nucleotide production from endothelial cells. Nitric Oxide (2015) 52:1–15. 10.1016/j.niox.2015.10.004 26529478 PMC4703509

[117] Koo A Nordsletten D Umeton R Yankama B Ayyadurai S García-Cardeña G In silico modeling of shear-stress-induced nitric oxide production in endothelial cells through systems biology. Biophys J (2013) 104(10):2295–306. 10.1016/j.bpj.2013.03.052 23708369 PMC3660651

[118] Kang HG Shim EB Chang K-S. A new multiphysics model for the physiological responses of vascular endothelial cells to fluid shear stress. J Physiol Sci (2007) 57(5):299–309. 10.2170/physiolsci.RP005407 17963593

[119] Lim YC Cooling MT Long DS. Computational models of the primary cilium and endothelial mechanotransmission. Biomech Model Mechanobiol (2015) 14(3):665–78. 10.1007/s10237-014-0629-x 25366114

[120] Nordgaard H Swillens A Nordhaug D Kirkeby-Garstad I Van Loo D Vitale N Impact of competitive flow on wall shear stress in coronary surgery: computational fluid dynamics of a LIMA-LAD model. Cardiovasc Res (2010) 88(3):512–9. 10.1093/cvr/cvq210 20581004

[121] McDougall SR Anderson ARA Chaplain MAJ. Mathematical modelling of dynamic adaptive tumour-induced angiogenesis: clinical implications and therapeutic targeting strategies. J Theor Biol (2006) 241(3):564–89. 10.1016/j.jtbi.2005.12.022 16487543

[122] Pontrelli G Halliday I Spencer TJ König CS Collins MW. Modelling the glycocalyx-endothelium-erythrocyte interaction in the microcirculation: a computational study. Comput Methods Biomech Biomed Engin (2015) 18(4):351–61. 10.1080/10255842.2013.799146 23734750

[123] Comerford A David T Plank M. Effects of arterial bifurcation geometry on nucleotide concentration at the endothelium. Ann Biomed Eng (2006) 34(4):605–17. 10.1007/s10439-005-9046-8 16568351

[124] Kar S Kavdia M. Modeling of biopterin-dependent pathways of eNOS for nitric oxide and superoxide production. Free Radic Biol Med (2011) 51(7):1411–27. 10.1016/j.freeradbiomed.2011.06.009 21742028 PMC3184605

[125] Kar S Kavdia M Endothelial NO and ${\text{O}}_{2}^{-}$ production rates differentially regulate oxidative, nitroxidative, and nitrosative stress in the microcirculation. Free Radic Biol Med (2013) 63:161–74. 10.1016/j.freeradbiomed.2013.04.024 23639567 PMC4051226

[126] Ghonaim NW Fraser GM Ellis CG Yang J Goldman D. Modeling steady state SO_2_-dependent changes in capillary ATP concentration using novel O_2_ micro-delivery methods. Front Physiol (2013) 4:260. 10.3389/fphys.2013.00260 24069001 PMC3781332

[127] Munaron L Scianna M. Multilevel complexity of calcium signaling: modeling angiogenesis. World J Biol Chem (2012) 3(6):121–6. 10.4331/wjbc.v3.i6.121 22905290 PMC3421110

[128] Wei XN Han BC Zhang JX Liu XH Tan CY Jiang YY An integrated mathematical model of thrombin-, histamine-and VEGF-mediated signalling in endothelial permeability. BMC Syst Biol (2011) 5(1):112. 10.1186/1752-0509-5-112 21756365 PMC3149001

[129] Sakellarios AI Papafaklis MI Siogkas P Athanasiou LS Exarchos TP Stefanou K Patient-specific computational modeling of subendothelial LDL accumulation in a stenosed right coronary artery: effect of hemodynamic and biological factors. Am J Physiol Heart Circ Physiol (2013) 304(11):H1455–70. 10.1152/ajpheart.00539.2012 23504178

[130] Nicolás M Peña E Malvè M Martínez MA. Mathematical modeling of the fibrosis process in the implantation of inferior vena cava filters. J Theor Biol (2015) 387:228–40. 10.1016/j.jtbi.2015.09.028 26458786

[131] Kagadis GC Skouras ED Bourantas GC Paraskeva CA Katsanos K Karnabatidis D Computational representation and hemodynamic characterization of in vivo acquired severe stenotic renal artery geometries using turbulence modeling. Med Eng Phys (2008) 30(5):647–60. 10.1016/j.medengphy.2007.07.005 17714975

[132] Cunnane EM Mulvihill JJE Barrett HE Walsh MT Simulation of human atherosclerotic femoral plaque tissue: the influence of plaque material model on numerical results. Biomed Eng Online (2015) 14(Suppl 1):S7 10.1186/1475-925X-14-S1-S7 25602515 PMC4306121

[133] Harris TB Launer LJ Eiriksdottir G Kjartansson O Jonsson PV Sigurdsson G Age, Gene/Environment Susceptibility-Reykjavik Study: multidisciplinary applied phenomics. Am J Epidemiol (2007) 165(9):1076–87. 10.1093/aje/kwk115 17351290 PMC2723948

[134] Holzinger ER Dudek SM Frase AT Krauss RM Medina MW Ritchie MD. ATHENA: a tool for meta-dimensional analysis applied to genotypes and gene expression data to predict HDL cholesterol levels. Pac Symp Biocomput (2013):385–96. 10.1142/9789814447973_0038 23424143 PMC3587764

[135] Metts B Thatcher S Lewis E Karounos M Cassis L Smith R DDDAS design of drug interventions for the treatment of dyslipidemia in ApoE(-/-) mice. J Dev drugs (2013) 2(2):107 10.4172/2329-6631.1000107 25866829 PMC4390998

[136] van Schalkwijk DB van Ommen B Freidig AP van der Greef J de Graaf AA. Diagnostic markers based on a computational model of lipoprotein metabolism. J Clin Bioinforma (2011) 1(1):29. 10.1186/2043-9113-1-29 22029862 PMC3305892

